# Cognitive functioning and mental health in children with a primary mitochondrial disease

**DOI:** 10.1186/s13023-022-02510-7

**Published:** 2022-10-01

**Authors:** Kim F. E. van de Loo, José A. E. Custers, Lonneke de Boer, Marloes van Lieshout, Maaike C. de Vries, Mirian C. H. Janssen, Christianne M. Verhaak

**Affiliations:** 1 Radboud Center for Mitochondrial Medicine, Department of Medical Psychology, Radboud Institute for Health Sciences, Amalia Children’s Hospital, Radboud University Medical Center, Geert Grooteplein Zuid 10, PO Box 9101, 6500 HB Nijmegen, The Netherlands; 2 Center for Neuropsychological Expertise, Denkkracht, Nijmegen, The Netherlands; 3 Radboud Center for Mitochondrial Medicine, Amalia Children’s Hospital, Radboud University Medical Center, Geert Grooteplein Zuid 10, PO Box 9101, 6500 HB Nijmegen, The Netherlands; 4 Centre of Excellence for Neuropsychiatry, Vincent Van Gogh Institute for Psychiatry, Venray, The Netherlands; 5 Radboud Center for Mitochondrial Medicine, Department of Internal Medicine, Radboud Institute for Molecular Life Sciences, Radboud University Medical Center, Geert Grooteplein Zuid 10, PO Box 9101, 6500 HB Nijmegen, The Netherlands

**Keywords:** Mitochondrial disease, Disease manifestation, Cognitive functioning, Attention, Working memory, Mental health, Quality of life

## Abstract

**Background:**

Studies regarding cognitive and mental health functioning in children with mitochondrial disease (MD) are scarce, while both are important issues given their impact on QoL. Knowledge on these aspects of functioning and its relationship with disease parameters is essential to gather more insight in working mechanisms and provide recommendations for future research and patientcare. The aim of this study was to map the cognitive functioning and mental health in children with MD in relation to disease specific factors.

**Methods:**

Pediatric patients (< 18 year) with a genetically confirmed MD were included. Demographic and disease specific factors (International Paediatric Mitochondrial Disease Scale) were assessed, as well as cognitive functioning (intelligence, attention, working memory (WM)), and mental health (psychological functioning and quality of life). Individual patient data was described.

**Results:**

Thirty-three children with MD were included. Intellectual functioning ranged from a clinically low IQ (36% of the patients, N = 12/33) to an average or above average IQ (39%, N = 13/33). A higher verbal versus performance IQ was observed (36% N = 5/14), a lower processing speed (43%, N = 6/14), attentional problems (50%, N = 7/14), and verbal WM problems (11%, N = 2/18). Regarding mental health, general behavioral problems were reported (45%, N = 10/22), and on subscale level, attention problems (45%, N = 10), withdrawn/depressed (36%, N = 8/22) and anxious/depressed behavior (14%, N = 3/22). Furthermore, QoL impairments were reported (42%, N = 5/12). The specific intelligence profiles, cognitive impairments, behavioral problems and QoL impairments occurred in every intelligence subgroup. Children with an average or above general intellectual functioning had a generally lower and less variability in IPMDS scores, less frequently epilepsy, vision and hearing problems, and a relatively later age of onset, as compared to patients with a clinically low intellectual functioning.

**Conclusions:**

Despite considerable heterogeneity, overall results showed a high rate of impairments in both cognitive and mental health functioning. Also in children with an average or above level of intellectual functioning, specific cognitive impairments were observed. Children with a clinically low intellectual functioning more often had disease related impairments compared to children with a higher intellectual functioning. The importance of structural assessment of cognitive functioning and mental health is warranted, also in children with mild disease related symptoms.

## Background

Primary mitochondrial diseases (MD) encompass a heterogeneous group of progressive disorders, with for each individual a unique and unpredictable trajectory [34]. Any tissue or organ can be affected in any combination, with a first presentation at any age [34]. As a consequence, there is an enormous complexity in diagnostics, treatment and prognosis of MD [34]. Due to the brain involvement in most patients with MD [4, 17, 31], and the link between mitochondrial dysfunction and mental health problems (e.g. [3, 27]), patients are vulnerable for impaired cognitive and mental health functioning. The unique pattern of disease is reflected in research on cognitive functioning and mental health in patients with MD: In general, considerable impairments on both domains are evident, but analysis of underlying subdomains shows clear heterogeneity regarding the details of the findings [14]. Importantly, most research in this area focuses on adults, and less is known about cognitive functioning and mental health in children with MD. Research in adults is not straightforwardly generalizable to children given their developmental nature, highlighting the importance to investigate cognitive functioning and mental health in children as well.

In children with MD, two studies reported on general intellectual functioning. Results showed a variety in functioning ranging from developmental delay to an average level of functioning [8, 36]. Both studies reported a negative relation of early disease onset and having seizures, with general intellectual functioning. In addition, a few case studies confirmed heterogeneity in cognitive functioning in these children, while also targeting specific cognitive domains such as attention, executive functioning and visual spatial functioning [19, 28, 29]. Interestingly, even in the context of similar genetic variants, cognitive profiles varied widely [28, 29]. Research focusing on adults reported also a variety in cognitive profiles, and deficits in visuo-spatial functioning, attention, executive functioning and memory, amongst other [14, 24]. Prerequisite for further understanding these differences in cognitive profiles in relation to disease specific measures is to systematically investigate different aspects of cognitive functioning in adults as well as in children with different types of MD.

Regarding mental health in children, two studies reported depressive behavior to be common in children with MD and suggested an association with abnormal central nervous system metabolism [15, 26]. Also behavioral problems, especially internalizing problems were reported [8], as well as a lower quality of life [21], and limitations in daily functioning [35]. The most burdensome symptoms reported in children are fatigue and a lack of energy, but also developmental delay and behavioral problems are frequently reported complaints [16]. Research on mental health and related disease specific factors is scarce and results are heterogeneous: For example, research in adults with MD showed that QoL is only partly reflected by clinical parameters and symptom status [45], while another study did not report any relation at all [32]. In summary, there is evidence for mental health problems in children with MD, however, there is limited research on related disease specific factors.

Knowledge on cognitive and mental health functioning and its relationship to biomedical parameters can contribute to provide optimized and targeted patient care. Systematical assessment of different aspects of functioning in which patients experience problems, amongst which their cognitive functioning and mental health, in relation to their disease manifestation, is essential. It can gather more insight in working mechanisms and provide recommendations for future research and patientcare. The aim of this study is to map the cognitive functioning and mental health in children with MD.

## Methods

### Participants and procedure

All pediatric patients (< 18 year) with a genetically confirmed mitochondrial disease (MD) and in follow-up at the department of Medical psychology of the Radboud Centre for Mitochondrial Medicine (RCMM) were included. Based on a clinical question patients were referred to the department of medical psychology by the pediatrician for (neuro)psychological assessment either (1) as part of a multidisciplinary admission called “mitoroute” within the RCMM (N = 31) and when indicated seen for additional assessment, or (2) referred directly based on a clinical question (N = 2). Forty-two patients were referred between 2014 and 2020, of which 33 patients (20 males, 13 females, ages 1–17 years) were included based on available psychological assessment. Of the remaining 8 patients, there was no data of cognitive functioning available (N = 2) or patients were not willing to participate (N = 6). Neuropsychological tests and questionnaires were included, to a varying extent depending on age of the child and the clinical question. In case a child participated more than once in the “mitoroute”, the most complete or extensive assessment was described. In case a specific test/ measure was only assessed in < 5 patients, the variable was excluded.

As part of standard care the study did not fall within the remit of the Medical Research Involving Human Subjects Act (WMO). All patients (> 12 years) and their parents provided informed consent.

### Materials

#### Disease specific factors

Inclusion criteria were a proven mitochondrial DNA or nuclear DNA variant(s) in known mitochondrial genes (see Table 3). Specific phenotypes were determined based on genotype and clinical presentation. The International Paediatric Mitochondrial Disease Scale (IPMDS), was used for measuring disease manifestation, by rating clinically relevant aspects of MD [50]. It contains 61 items in three domains: (1) subjective symptoms and complaints (23 items based on interviewing parents), (2) physical examination (25 items), and (3) functional abilities (13 items obtained by physical/motor function evaluation). Furthermore the following factors were included: epilepsy (yes/no), hearing problems (yes/no), vision problems (yes/no), motor disabilities (yes/no).

#### Cognitive functioning

1. *Intelligence*
  Intellectual functioning was assessed by validated, age appropriate tests. Children younger than 2;6 years, or with an estimated mental retardation were assessed with the Dutch versions of the Bayley Scales of Infant Development- Second Edition (BSID-II-NL) [42], or Third Edition (Bayley-III-NL) to provide a developmental index [40]. Depending on the age of the child, different versions of the Dutch versions of the Wechsler intelligence test were used,the Wechsler Preschool and Primary Scale of Intelligence-Third Edition (WPPSI-III-NL) [49], the Wechsler Intelligence Scale for Children-Third Edition (WISC-III-NL) [18], or the updated version, the WISC-V-NL [48], and the Wechsler Adult Intelligence Scale (WAIS-IV-NL) [47]. Full Scale Intelligence Quotient (FSIQ) was reported, Verbal Intelligence Quotient (VIQ), Performance Intelligence Quotient (PIQ) (WISC-III-NL) (or in case of WISC-V-NL, the Visual Spatial Index), and Information Processing Speed Factor (WISC-III-NL) (PSF) (or in case of WISC-V-NL, the Processing Speed Index). In case children were only seen for screening in the multidisciplinary “mitoroute”, patients were screened with four subtests of the intelligence test (*Information*, *Vocabulary*, *Block Design*, and *Object Assembly* (WISC-III-NL) or *Matrix Reasoning* (WPPSI-III-NL)). In this case the Full Scale IQ (FSIQ) was estimated based on proration by four subtests named as FSIQ_est_ [22]. The subtests correlated between 0.59 and 0.75 with the FSIQ for the WISC-III [18], and between 0.64 and 0.72 with the FSIQ for the WPPSI-III-NL [49]. Children were tested with a non-verbal intelligence test, the Snijders-Oomen Non-verbal Intelligence test (SON-R), in case of hearing or language problems [37]. VIQ versus PIQ scores were calculated and in case of a difference of ≥ 15 IQ points interpreted as significant, consistent with previous research (e.g. [5, 13]. In the same line, the PSF was interpreted as deviant from the other indexes in case of a difference of ≥ 15 IQ points.
2. *Attention*
  Attention was measured by three auditory attention tests of the Test of Everyday Attention for Children (TEA-Ch) for children aged 6–16 years [20]. (1) In the sustained attention test “*Score!”*, patients had to silently count the number of tones (9–15 tones) presented in varying intervals, and announce the total number at each end of the trial (10 in total). (2) In the ‘dual test’ for divided attention, “*Score DT”*, patients had to listen to specific targets among distracters. Patients had to count the number of tones while listening to a new broadcast and naming the animal mentioned in it (10 trials in total). (3) In the sustained attention test “*Code Transmission”* patients only had to respond to a target when this was proceeded by a specific cue. Patients had to listen to a long monotone series of digits and immediately announce the digit presented before ‘5 5’ when they heard these numbers (40 targets in total). In case it was visible/ observable or on comment of the child that the speed of presentation was too high, the test was stopped. In all tests, the total number of correct trials/ targets was scored. The tests had an age based normative mean of 10 (SD = 3). See for more details about the description and validation Underbjerg et al. [39] and Heaton et al. [10].
3. *Working memory*
  Subtests of the Kaufman Intelligence test were used to assess verbal and non-verbal/visual working memory (WM) for children 4–16 years [12]. In the non-verbal/visual test, “*Hand Movements”*, patients had to repeat various movements of the hands, increasing in number of movements. In the verbal test, “*Number Recall”*, patients had to repeat digits in the same order as presented, increasing in the number of digits. The tests had an age based normative mean of 10 (SD = 3).

Scores regarding cognitive functioning were interpreted as follows:

#### Mental health

1. *Psychological functioning*
  Psychological functioning of the child, in terms of behavioral problems, was assessed with the Child Behavior Checklist (CBCL) [1, 2]. The CBCL is a parent-reported questionnaire that provides scores on behavioral problems, divided into two age categories: 1.5 to 5 years (99 items) and 6 to 18 years (113 items) [46]. Based on clinical concerns and existing literature in MD patients [26],van [41] scales of interest were a priori selected. The Total scale, for assessment of global behavioral problems, and in addition the subscales anxious/depressed behavior, withdrawn/depressed behavior and attention problems were included. Scores were rated as ‘normal’ (Total T-scores ≤ 59, subscale T-scores ≤ 63), ‘borderline’ (Total T-scores ≥ 60–63, subscale T-scores ≥ 64–69), or ‘clinical’ (Total T-score ≥ 64, subscale T-scores ≥ 70) [1, 2].
2. *Quality of life*
  Quality of life (QoL) was assessed with the Pediatric Quality of Life Inventory (PedsQL) [44]. Parents of children from 5 to 13 years old reported on the general quality of life of the child. The PedsQL consists of 23 items divided into five subscales: Physical (8 items), Emotional (5 items), Social (5 items), and Scholar functioning (5 items). Answering options were on a 5-point Likert scale: never (0), almost never (1), sometimes (2), often (3), and almost always (4). Each answer was reversed scored and rescaled to 0–100 scale (0 = 100, 1 = 75, 2 = 50, 3 = 25 and 4 = 0). A higher score indicates a better QoL [43].

### Statistical analysis

Individual tests were scored according to the test manuals as described above. In case a test was too difficult, or a test could not be performed due to severe vision/ hearing/ or motoric problems, or in case of visible unreliable results, the test was stopped and not included in analyses. Due to the large heterogeneity in MD diagnoses, as well as diversity in tests used (e.g. due to different age versions, physical limitations, mental retardation), we decided to describe individual patient data instead of performing analyses on group level. To investigate differences between children based on their general intellectual functioning, we defined subgroups (see Table 1): *average or above IQ* ((estimated) FSIQ ≥ 80), *borderline IQ* ((estimated) FSIQ 70–79), and a *clinically low IQ* (children with an (estimated) FSIQ or developmental functioning of ≤ 69). We additionally compared if there was a ‘match’ in results on the attentional test(s) (TEA-Ch) and reported attention problems (CBCL) by defining problems on either one of these measures as a borderline or clinical score.

**Table 1:** Classification of scores

IQ-score	Normscore	Classification^	Subgroups for analyses
≥ 130	≥ 16	Exceptionally high	Average range or above
129–120	15–14	Above average	
119–110	13–12	High average	
109–90	11–9	Average	
89–80	8–6	Low average	
79–70	5–4	Below average	Borderline
≤ 69	≤ 3	Exceptionally Low	Clinically low

^Classification of scores based on Guilmette et al. [51] and Hendriks et al. [52]

## Results

Thirty-three children with a diagnosed MD were included, of which most patients (N = 10) had the mDNA 3243 A > G variant in mitochondrial DNA. For the remaining variants, phenotypes, patient characteristics and specific test results see Table 2 and 3.

**Table 2:** Cognitive functioning and mental health (n = 33)

Patient number	Mutation	Gender	Age	education	Dev. age in months	FSIQ^7^	VIQ^7^	PIQ/ VRI^7^	PSF/ PSI^7^	Score!^8^	Score DT^8^	Code transimission^8^	Verbal WM^8^	Visual WM^8^	Behavioral problems Total^9^	Anxious/depressed^9^	Withdrawn/ depressed^9^	Attention problems^9^	QoL^10^
*Average or above IQ (IQ* ≥ *80; n* = *13)*																			
1	mDNA 3243 A > G	M	10	S		121^3^	129	105	65	8	5	7	9	10	62	57	70	59	77,17
2	mDNA 3243 A > G	M	16	R		121^5^	113	125	97										
3	mitochondrial deletion, 9101_14603del	F	16	R		123^3^*				8	6	2	7	9	71	80	89	68	61,96
4	mDNA 3242 A > G	F	9	R		120^3^*													
5	mitochondrial deletion, 8470_13446del	M	13	R		120^3^*				7	5	7	8	12	46	50	57	50	70,65
6	mitochondrial deletion, 8649_16084del	M	11	R		118^4^	130	108	100	11	14				57	50	58	64	
7	mDNA 3243 A > G	M	15	R		116^3^*				7	7	10	8	12	63	57	85	53	50
8	NDUFS7, c.364G > A, (p.Val122Met), homozygous	F	5	R		110^2^*							8	10					
9	mDNA 04,300 A > G	M	5	R		92^2^	87	100	94				6	7	70	64	70	57	
10	mDNA 8993T > G	M	7	R		87^2^	110	85	55	2			6	8					
11	mDNA 3243A > G	M	13	R		84^4^	78	92	83	11	2				51	54	53	53	65,22
12	FXN homozygous expansion repeats	M	11	R		84^3^*				11	11	11	8	10	42	50	50	50	64,13
13	mDNA 4404T > C	F	10	R		84^3^*													39,13
*Borderline IQ (IQ* = *70–79; n* = *8)*																			
14	mDNA 3243 A > G	M	11	R		78^3^	88	72	67	9	8	5	11	11	60	57	58	61	
15^†^	mDNA 3243 A > G	M	12	S		78^3^	86	73	67	9	5	TD	8	9	55	54	57	53	
16	mDNA 7507 A > G	M	7	S		78^3^*				TD	TD	TD	6	11	73	57	66	66	43,48
17^†^	SURF1, c.312_321delinsAT, (p.Leu105*), homozygous	F	2	-	-6	77^1^													
18^†^	RRM2B, 1045 G > A, (pAla349Thr), homozygous	F	1	-	-3	72^1^													
19	NDUFS1, c.683T > C, (p.Val228Ala), 737 + 3 A > G (spl?)	F	15	S		71^3^	73	75	83	8	2	6	6	9	80	82	78	83	27,17
20	mDNA 3243 A > G	F	16	R		70^3^*				10	11	10	11	10	69	59	60	63	45,65
21	mDNA 09185T > C	M	8	R		70^3^	76	68	75	3	1	2	5	8	41	50	50	51	58,7
*Clinically low IQ (IQ* < *70; n* = *12)*																			
22	mDNA 3243 A > G	F	12	S		62^3^	79	55	67	4	2	1	6	7	58	55	51	70	
23	mDNA 3243 A > G	M	16	S		58^3^	63	58	55				1	5					
24	FBXL4 c.1641_1642del (p.Cys547*), c.1252G > C (p.Ala418Pro)	F	7	S		55^6^									62	52	52	77	
25	SDHA c.1535G > A (p.Arg512Gln), c.1753C > T (p.Arg585Trp)	M	4	S		< 55^2^	< 55	55							69	56	76	77	
26	SDHA, c.64-2A > G, splicing defect homozygous	M	11	S		50^6^									43	50	56	70	
27	RARS2, c.442A > G (p.Thr148Ala), c.1519G > A (p.Glu507Lys)	M	17	S		46^3^	< 55	< 55	< 55				1	5	59	57	78	64	
28	RARS2, c.442A > G (p.Thr148Ala), c.1519G > A (p.Glu507Lys)	F	12	S		46^3^*				1	1		4	5	56	50	57	68	
29	OPA1, c.1156_1157del (pLeu386Glufs*2)	F	3	-	-18										46	50	56	62	
30	FBXL4, c.1361A > C (p.Gln454Pro), homozygous	M	6	S	-33														
31	SUCLA2 1271delG9p (pGly424Aspfs*18), homozygous	M	4	S		NA^													
32	ACO2 2012G > A (pArg671Gln), 2050C > T(pArg684Trp)	F	8	S		NA^									51	50	52	52	63,04
33^†^	NDUFS7 c.364G > A(pVal122Met) homozygous	M	14	S		NA^													

*Estimated IQ based on 4 subtests, ^1^BSID-II-NL/Bailey-III, ^2^WPPSI-III-NL, ^3^WISC-III-NL, ^4^WISC-V, ^5^WAIS-IV, ^6^SON-R, ^7^IQ scores, ^8^Normscores, ^9^T-scores, ^10^scaled scores (0–100)

^Not tested due to severe developmental problems or severe developmental and vision hearing/motoric problems

TD, too difficult

†Died during the course of the study

**Table 3:** Demographics and disease characteristics

Patient number	Mutation	Level of heteroplasmy*	Clinical syndrome	Gender	Age	Age of onset	Motor disability	Vision problems	Hearing problems	Epilepsy	IPMDS score
*Average or above IQ (IQ* ≥ *80; n* = *13)*											
1	mDNA 3243 A > G	81% (urine)		M	10	10 y	1	0	0	0	13
2	mDNA 3243 A > G	85% (urine)		M	16	17 y	1	0	0	0	NA
3	mitochondrial deletion, 9101_14603del		CPEO	F	16	14 y	0	0	0	0	12
4	mDNA 3242 A > G	71% (urine), 53% (cheek swab)		F	9	9 y	0	0	0	0	12
5	mitochondrial deletion, 8470_13446del		CPEO	M	13	5 y	1	0	0	0	NA
6	mitochondrial deletion, 8649_16084del	35% (muscle)	CPEO	M	11	3 y	1	0	0	0	5
7	mDNA 3243 A > G	46%		M	15	10 y	1	0	0	0	NA
8	NDUFS7, c.364G > A (p.Val122Met), homozygous		Leigh	F	5	2 y	1	0	0	0	NA
9	mDNA 04,300 A > G	73%		M	5	18 mo	0	0	0	0	NA
10	mDNA 8993T > G	99%	Leigh	M	7	18 mo	1	0	0	0	NA
11	mDNA 3243 A > G	47%	MELAS	M	13	12 y	0	0	0	1	11
12	FXN homozygous expansion repeats		Friedreich’s ataxia	M	11	5 y	0	0	0	0	NA
13	mDNA 4404T > C	11%, 96% (muscle)		F	10	9 y	1	0	0	0	32
*Borderline IQ (IQ* = *70–79; n* = *8)*											
14	mDNA 3243 A > G	75% (muscle) 96% (urine)		M	11	11 y	1	0	0	0	NA
15^†^	mDNA 3243 A > G	90% (muscle)	MELAS	M	12	9 y	0	0	0	0	NA
16	mDNA 7507 A > G	97%		M	7	2 y	1	1	1	0	NA
17^†^	SURF1, c.312_321delinsAT (p.Leu105*), homozygous		Leigh	F	2	first year	1	0	0	0	NA
18^†^	RRM2B, 1045 G > A (pAla349Thr), homozygous			F	1	2 mo	1	0	1	0	NA
19	NDUFS1, c.683T > C, (p.Val228Ala), 737 + 3A > G (spl?)			F	15	14 y	1	1	0	0	NA
20	mDNA 3243 A > G	40%		F	16	14 y	0	0	0	0	NA
21	mDNA 09,185T > C		NARP	M	8	6 y	1	1	1	0	NA
*Clinically low IQ (IQ < 70; n = 12)*											
22	mDNA 3243 A > G	65%		F	12	10 y	1	0	0	0	16
23	mDNA 3243 A > G	87% (muscle)	MELAS	M	16	10 y	1	0	1	1	NA
24	FBXL4 c.1641_1642del (p.Cys547*), c.1252G > C (p.Ala418Pro)			F	7	first year	1	0	0	0	NA
25	SDHA c.1535G > A (p.Arg512Gln), c.1753C > T (p.Arg585Trp)			M	4	6 mo	1	0	0	1	9
26	SDHA, c.64-2A > G, splicing defect homozygous			M	11	15 mo	1	0	0	0	63
27	RARS2, c.442A > G (p.Thr148Ala), c.1519G > A (p.Glu507Lys)			M	17	9 mo	1	0	0	0	NA
28	RARS2, c.442A > G (p.Thr148Ala), c.1519G > A (p.Glu507Lys)			F	12	first year	1	0	0	0	NA
29	OPA1, c.1156_1157del (pLeu386Glufs*2)		DOAplus	F	3	8 mo	1	1	0	1	NA
30	FBXL4, c.1361A > C (p.Gln454Pro), homozygous			M	6	after birth	1	1	1	1	NA
31	SUCLA2 1271delG9p (pGly424Aspfs*18), homozygous			M	4	5 mo	1	1	1	0	96
32	ACO2 2012G > A (pArg671Gln), 2050C > T(pArg684Trp)			F	8	14 mo	1	1	0	0	37
33^†^	NDUFS7 c.364G > A(pVal122Met) homozygous			M	14	2 y	1	0	0	0	134

*Blood or specified in which other tissue

^†^Died during the course of the study

### Cognitive functioning

Regarding *general intellectual functioning*, 39% scored in the average range or above (N = 13/33), 24% in the borderline range (N = 8), and 36% in the clinically low range (N = 12) (see Table 2). 45% of the children followed regular education (N = 15/33), 45% special education and three patients did not attend school yet due to their age. All, except one child with an *average or above IQ,* followed regular education. Specific subgroup analyses of the three intelligence groups revealed the following:

*Average or above IQ range*: Six out of thirteen patients completed a full intelligence test, of which three had a higher verbal compared to performance IQ, and four out of these six a lower processing speed compared to their verbal and/or performance IQ scores. Three out of eight patients had clinical scores on one of the attention tests, and one patient borderline scores. Three patients scored in the average range on the attention tests, however one of them had low average scores. Regarding working memory (WM), no patients had clinical or borderline scores, however low average scores occurred more frequently in verbal WM compared to visual WM (Verbal WM 7/8 patients, visual WM 2/8 patients).

*Borderline IQ range*: Four out of eight patients completed a full intelligence test, of which one had a higher verbal compared to performance IQ, and two patients a lower processing speed compared to their verbal IQ scores. Six out of eight patients performed attentional tests. For two patients, one or more of these tests were too difficult. These specific administrations were ended and not taken into account. Of the other four patients, two had clinical scores and one borderline scores on one or more of the attention tests, leaving out one patient with no attention problems. On both the verbal and visual WM there were no patients with clinical scores, only one out of the six tested patients had a borderline score on verbal WM.

*Clinically low IQ range*: Out of the twelve patients in this clinically low IQ range, nine were classified as having an ‘exceptionally low IQ/ developmental functioning’ and three were ‘not testable’ regarding their general cognitive functioning. These three patients could not be tested due to severe developmental problems or severe developmental and visual/ hearing/ motor problems, and all had an estimated IQ < 69. Four out of the nine tested patients completed a full intelligence test, of which one (out of four) had a higher verbal compared to performance IQ. Two out of the nine tested patients performed attention tests; all scores were in the borderline to clinical range. Four out of nine patients performed both working memory tasks; two out of these four patients had clinical scores and one borderline scores on verbal WM, none of the patients had clinical scores on visual WM, however three in the borderline range.

In summary, intellectual functioning ranged from an exceptionally low IQ to an above average IQ. One third of the patients had an average or above intellectual functioning. Overarching, results showed verbal versus performance IQ differences in 36% (N = 5/14), and lower processing speed compared to verbal and/or performance IQ in 43% (N = 6/14), as well as attentional problems (clinical scores) in 50% of the patients (N = 7/14), verbal WM problems in 11% (N = 2/18) and no visual WM problems. These specific intelligence profiles and cognitive impairments occurred in every intelligence subgroup.

### Mental health

In twenty-two out of the thirty-three patients information regarding behavioral problems was available. *General behavioral problems* (Total scale) were observed in 45% of the patients (N = 10/22) (including both borderline and clinical scores), leaving out 55% with no behavioral problems in general. In addition, on a subscale level, in 45% of the patients (N = 10/22) attention problems were reported (including both borderline and clinical scores), in 36% (N = 8/22) withdrawn/ depressed behavior, and in 14% (N = 3/22) anxious/ depressed behavior.

In total, 15 patients performed both attentional test(s) and filled in the behavioral questionnaire, however 1 patient was excluded for analyses because the attentional tests were too difficult. There was a match in results in 50% of the patients; either attentional problems tested and reported on behavioral level (N = 4/14), or no problems on both (N = 3/14). However, in 43% of the patients (N = 6/14) attentional problems were established based on cognitive test results, but were not reported on the behavioral level. In one patient, neurocognitive testing revealed no attentional problems, while these problems were reported on the behavioral level.

Specific subgroup analyses revealed (borderline and clinical) general behavioral problems in 50% (N = 4/8) of the patients with an *average or above IQ*. Additionally, on the subscale level withdrawn/ depressed behavioral problems were most frequently reported (N = 4), followed by attentional problems and anxious/ depressed behavior (both N = 2). In 66.7% (N = 4/6) of the patients with a *borderline IQ*, (borderline and clinical) general behavioral problems were reported. Additionally, on the subscale level attention problems were most common (N = 3), followed by withdrawn/depressed behavior (N = 2) and anxious depressed behavior (N = 1). In 25% (N = 2/8) of the patients with a *clinically low IQ*, (borderline and clinical) general behavioral problems were reported. Additionally on the subscale level 75% (N = 6) reported attentional problems, two patients withdrawn/depressed behavior, and none anxious/depressed behavior.

For twelve out of the thirty-three patients information regarding QoL was available. In total, 42% of these patients (N = 5/12) had a lower *QoL* in the borderline to clinical range. Four of them also reported behavioral problems, and the remaining one did not fill in the behavioral questionnaire. Subgroup analyses revealed a lower QoL in 29% of the patients with an *average or above IQ* (N = 2/7), 75% of the patients with a *borderline* IQ (N = 3/4), and no impairments in the one patient tested with a *clinically low IQ*.

In summary, in almost half of the patients general behavioral problems were reported. On subscale level, attention problems were most frequently reported, followed by withdrawn/ depressed and anxious/depressed behavior. Behavioral problems in general and on subscale level occurred in every intelligence subgroup. QoL impairments were reported in 42%, though only for a small sample of patients these data was available.

### Disease specific outcomes

In total 79% (N = 26/33) patients had motor disabilities, 21% vision problems, 18% hearing problems and 15% epilepsy. Subgroup analyses revealed that out of the 13 children with an *average or above IQ*, 62% (N = 8/13) had motor disabilities, one had epilepsy, and none had vision or hearing problems. Of the 8 children with a *borderline IQ*, 75% (N = 6/8) had motor disabilities, 37.5% had vision problems, 37.5% hearing problems, and none had epilepsy. Of the children with a *clinically low IQ*, all had motor disabilities (N = 12), 33% vision problems, 25% had hearing problems and 33% had epilepsy.

International Paediatric Mitochondrial Disease Scale (IPMDS) scores were assessed in 12 patients. In children with an *average or above IQ,* IMPDS scores varied between 5 and 32 (N = 6), while IPMDS scores of children with a *clinically low IQ* varied between 9 and 134 (N = 6). In the subgroup of children with a *borderline IQ,* no IPMDS scores were available. Age of onset varied between expression at birth and 14 years of age. Children with a *clinically low IQ* more often had an age of onset within the first two years of life (83%, N = 10/12), compared to children with a *borderline* (38%, N = 3/8) and *average or above IQ* (33%, N = 5/15). Four of all 33 (12%) children died, with ages between 1 and 14 years. These four children all had an intellectual/ developmental functioning in the *borderline* or *clinically low IQ range*. Five out of the 10 patients with mDNA3243 A > G variant (50%), had an *average or above* IQ, and three out of the 10 patients (30%) a *borderline IQ*.

In summary, results showed that children with an average or above general intellectual functioning had a generally lower score and less variability in IPMDS scores, less frequently epilepsy, vision and hearing problems, and relatively later age of onset, as compared to patients with a clinically low intellectual functioning.

## Discussion

Despite considerable heterogeneity, overall results showed a high rate of impairment in both cognitive and mental health functioning. In half of children with an average or above level of general intellectual functioning, specific cognitive impairments, amongst which attention problems, were observed. Children with a clinically low level of intellectual functioning more often had disease related impairments compared to children with a higher level of intellectual functioning.

Results showed a general intellectual/developmental functioning ranging between an exceptionally low to an above average level, and a specific intelligence profile in a substantial amount of children; a higher verbal versus performance IQ and/or a lower information processing speed compared to either the verbal or performance IQ. The relatively lower performance IQ and processing speed are not likely to be explained by the motor speed component of both, since previous research did not report motor speed deficits/deficits in simple reaction time tasks [6, 23]. Furthermore, another study reported processing speed impairments, after correction for motor speed difficulties [25]. It is suggested therefore, that these impairments in performance IQ and processing speed might be caused by other cognitive deficits, e.g. deficits in cognitive processing speed, visual analysis, visuo-spatial perception, problem solving skills and/or attention/concentration.

Results of the current study suggest a vulnerability for attention, and to a lesser extent (mainly verbal) WM problems in children with MD, which cannot solely be explained by a more global cognitive deficit. Remarkably, in all ranges of intellectual functioning, attentional deficits were apparent, at least in some of the children. Altogether, half of the children showed attention problems (50%), including sustained and divided attention. Attention problems have been previously reported in adult literature (see [14] for an overview). Verbal working memory (WM) problems occurred to a lesser extent (11%), consistent with findings in adult patients [38], while no clinical visual WM deficits were observed. When interpreting these results, it is important to note that a ‘deficit’ was scored as compared to chronological age based normative data, instead of compared to their general level of intellectual abilities, in which impairments are rated as compared to the individuals’ general level of intellectual functioning. When analyzing scores compared to patients own individual level of intellectual abilities, results showed lower than expected scores in both attention and verbal WM in almost all patients with an average or above level of intellectual functioning. Attention and working memory problems are known to have a significant impact on school performance, even in children with an average intellectual functioning (e.g. [7, 9, 30]). This indicates that, even in the relatively ‘well’ functioning patients, one should be aware of these relative weaknesses.

Regarding mental health, in more than half of the patients behavioral problems were reported, and in 42% a lower QoL. These problems were reported independent of general intellectual level of functioning. In line with previous literature, withdrawn/depressed behavior was reported [8, 26], as well as attention problems and to a lesser extent anxious/depressed behavior. In half of the patients, results of the attention test were in agreement with the behavioral report of attention problems. Though both measure different constructs, namely attention on a cognitive level versus behavioral attention problems, it is remarkable that there were more frequently attentional problems established on the cognitive level than were reported on the behavioral level by their parents. These results underline the importance of cognitive tests and indicate that neuropsychological testing is more sensitive to detect cognitive deficits compared to clinical assessment [11].

In relation to disease specific outcomes, results showed that children with an average or above level of general intellectual functioning had a generally lower score and less variability in IPMDS scores, less frequently epilepsy, vision and hearing problems, motor disabilities, and a relatively later age of onset, as compared to children with a clinically low level of intellectual functioning. Although there are marked individual differences, results furthermore indicate that the relatively ‘well’ functioning patients (as in average or above intellectual abilities with relatively less clinical signs and symptoms) are nonetheless prone to specific cognitive deficits and/or mental health problems.

To our knowledge, this is the first study investigating children with MD in multiple areas, i.e. in terms of cognitive functioning, mental health and the relation to disease specific outcomes. Another strength is the relatively large sample size, especially in light of the rareness of this disease. Finally, another strength is that data were obtained from regular patient care, and patients did not experience any additional burden. However, a downside of this method is that not all data were available for all children. As a consequence, for example in the clinically low intelligence group, less children performed additional cognitive tests and only one QoL questionnaire was available, while in the other intelligence groups, more data was available. This limits firmer conclusions. Another consequence of this limited data was that we did not specify particular attention deficits (i.e. divided, or sustained attention) but we more generally interpreted a lower score on either one of the three auditory attention tests as ‘attention deficit’. Furthermore, unfortunately, we only had access to the data of the hospital in which this research was part of. Other clinical details, like number of admissions to the hospital, is important information as a parameter for disease manifestation, and also because of the impact on QoL. Due to the fact that in some cases, children were also seen in other regional hospitals, mostly more nearby there home, we did not had access to all data. To avoid bias, we choose not to include this measure. Another limitation is that children were referred to the medical psychology department based on a clinical question or as part of a standard multidiscipline care program. Due to the fact that only 2 children were referred based on a clinical question, a possible bias seems less likely. All in all, this study provides a robust first step in research on cognitive functioning and mental health in relation to disease specific measures in children with MD.

For future research, we recommend longitudinal natural history studies, assessing children systematically as part of regular care from start of diagnosis on multiple domains including both cognitive functioning and mental health in addition to disease specific outcomes. For future research it is recommended to investigate multiple cognitive domains, in order to identify specific cognitive deficits, given te heterogeneity in MD. Furthermore, given the enormous heterogeneity in MD, a large sample size is necessary for research. Collaboration between different centers of expertise can be an important step for future research.

In conclusion, the importance of structural assessment of cognitive functioning and mental health is warranted, also in children with mild disease related symptoms. For patient care, it is highly recommended to assess patients for cognitive and mental health problems. More specifically, based on results of this study we recommend:

- Neuropsychological assessment, including a complete intelligence test, given the strengths and weaknesses within intelligence profiles as underlined by this study and previous literature. Validated tests, like the Weschler intelligence tests, are recommended.
- Assessment of attention: Results of this study underline testing of attention.
- Regarding mental health, screening for withdrawn/ depressed and attentional behavioral problems is recommended.
- Given the high QoL impairments in MD, it is important for clinicians to screen for and to be aware of these impairments: Actively ask for, and act on outcomes.

In line with the patient care standards [33] we recommend baseline neuropsychological testing and repeating of testing in case of cognitive, behavioral, or personality change and for following of their function and development. Furthermore, routinely screening for mental health problems is advised. Especially in children, given their developmental nature, it is important to repeat neuropsychological testing as well as screening for mental health problems, when transitioning between developmental phases like going to school, starting puberty or leaving secondary education. These transition periods generally challenge resilience for children with specific vulnerabilities. We recommend that a psychologist should be part of the multidisciplinary team and also in the transition clinic. Structural assessment of both cognitive and mental health functioning is important in order to provide adequate help in an early phase and to prevent them for escalation.

## Data Availability

The datasets analyzed during the current study are available from the corresponding author on reasonable request.

## Abbreviations

MD: Mitochondrial disease
RCMM: Radboud Center for Mitochondrial Medicine
QoL: Quality of life
IPMDS: International Paediatric Mitochondrial Disease Scale
BSID-II-NL: Bayley Scales of Infant Development-Second Edition
Bayley-III-NL: Bayley Scales of Infant and Toddler Development-Third Edition
WPPSI-III-NL: Wechsler Preschool and Primary Scale of Intelligence-Third Edition
WISC-III-NL: Wechsler Intelligence Scale for Children-Third Edition
WISC-V-NL: Wechsler Intelligence Scale for Children-Fifth Edition
SON-R: Snijders-Oomen Non-verbal Intelligence test
WAIS-IV-NL: Wechsler Adult Intelligence Scale
FSIQ: Full Scale Intelligence Quotient
VIQ: Verbal Intelligence Quotient
PIQ: Performance Intelligence Quotient
TEA-Ch: Test of Everyday Attention for Children
WM: Working memory
PedsQL: Pediatric Quality of Life Inventory
CBCL: Child Behavior Checklist
